# Correlation Between Tumor Necrosis Factor-α Levels, Free Fatty Acid Levels, and Soluble Vascular Cell Adhesion Molecule-1 Levels in Rheumatoid Arthritis Patients

**DOI:** 10.2174/1874312901812010086

**Published:** 2018-07-19

**Authors:** Fazria Nasriati, Rudy Hidayat, Budiman Budiman, Ikhwan Rinaldi

**Affiliations:** 1 Department of Internal Medicine, Faculty of Medicine Universitas Indonesia -s Cipto Mangunkusumo General Hospital, Jalan Diponegoro 71,Jakarta 10310,Indonesia; 2 Division of Rheumatology, Department of Internal Medicine, Faculty of Medicine Universitas Indonesia - Cipto Mangunkusumo General Hospital, Jalan Diponegoro 71,Jakarta,Indonesia; 3 Division of Endocrinology and Metabolic Diseases, Department of Internal Medicine, Faculty of Medicine Universitas Indonesia - Cipto Mangunkusumo General Hospital, Jalan Diponegoro 71,Jakarta,Indonesia; 4 Clinical Epidemiology Unit, Department of Internal Medicine, Universitas Indonesia - Cipto Mangunkusumo General Hospital, Jalan Diponegoro 71,Jakarta,Indonesia

**Keywords:** Tumor Necrosis Factor-α, Free Fatty Acids, Vascular Cell Adhesion Molecule-1, Rheumatoid Arthritis, SLE, ELISA, SLE, FFA

## Abstract

**Background:**

The mortality of Rheumatoid Arthritis (RA) is quite high, which is largely due to cardiovascular complications caused by endothelial dysfunction. One of the important inflammatory mediators that contribute to RA joints arthritis of TNF-α, also proven to play a role in endothelial dysfunction and play a role in increasing intracellular lipolysis, thus increasing circulating FFA levels.

**Objectives:**

To determine the correlation between TNF-α levels with VCAM-1 levels, correlation of TNF-α levels with FFA levels, and correlation of FFA levels with VCAM-1 levels.

**Methods:**

Cross sectional and retrospective design studies of adult RA patients treated at Cipto Mangunkusumo Hospital (RSCM), without metabolic disturbances, acute infection, cardiovascular disorders, or other autoimmune diseases. The cross-sectional data was collected from October to November 2017, while retrospective samples were collected since August 2016. TNF-α, VCAM-1, and FFA levels were assessed by serum blood test by ELISA method. Correlation analysis is done by Pearson analysis when the data distribution is normal and with Spearman analysis when the data distribution is not normal.

**Results:**

A total of 35 subjects were enrolled in the study. Most (97.1%) were women with an average age of 45.29 years, median disease duration of 48 months, and most had moderate disease activity (65.7%). No significant correlation was found between TNF-α levels and VCAM-1 levels (p = 0.677; r = +0.073). as well betwen TNF-α levels and FFA levels (p = 0.227; r = -0.21). The correlation between FFA and VCAM-1 levels showed significant correlation with negative correlation and weak correlation (p = 0.036; r = -0.355).

**Conclusions:**

(1) There was no correlation between TNF-α levels and VCAM-1 levels in RA patients; (2) There was no correlation between TNF-α levels and FFA levels in RA patients; (3) There was a negative correlation between FFA levels and VCAM-1 levels in RA patients.

## 1 INTRODUCTION

Rheumatoid Arthritis (RA) is one of the chronic autoimmune diseases with an estimated prevalence of 1 to 2 percent of the total population in the world [1]. The mortality of the disease is quite high, which is largely due to earlier cardiovascular complications [2] Cardiovascular risk is mainly due to the atherosclerosis process initiated by endothelial dysfunction and recorded 2 times greater in RA patients than in the general population [3, 4]. Endothelial dysfunction itself is a process that involves various factors, such as genetic, traditional cardiovascular risk factors, and systemic inflammation [3].

Del Rincon *et al.* in his prospective study of 236 RA patients over 8 years, reported that the cardiovascular risk in RA patients was 3.96 times compared to controls (95% CI 1.86-8.43). After being adjusted for traditional risk factors, this relative risk only decreased into 3.17 times compared to controls (RR 3.17 [95% CI 1.33-6.36]). The differences of RR obtained in this study showed that traditional risk factors are not the most important factor in cardiovascular events in RA patients, but other mechanisms may involved in the pathogenesis of RA disease itself, *i.e.* systemic inflammation [5].

Systemic inflammation that occurs in RA patients involves various pro inflammatory mediators, especially Tumor Necrosis Factor-α (TNF-α) [3] The role of TNF-α against endothelial dysfunction is evidenced by an experimental study conducted by Goodwin *et al.* (2007) on endothelial aortic cell cultures, where TNF-α lowered nitric oxide (NO) production by endothelial cells [6]. The effects of TNF-α on endothelial dysfunction and cardiovascular events in RA patients were then demonstrated in a study by Rho *et al.* which found a significant association between TNF-α (OR = 1.49, 95% CI (1.16-1.90)) with coronary calcium levels [7]. Meanwhile, the direct effect of TNF-α on endothelial dysfunction markers in RA patients was showed by Foster *et al.* (2009), where there was a strong correlation between TNF-α and VCAM (r = 0.322, P = 0.009) [8].

TNF-α is also one of the cytokines that have the ability to regulate various biological processes such as energy metabolism [9]. Study of Kawakami *et al.* (1987) demonstrated the decreased of lipopolysaccharide activity and increased of intracellular lipolysis of rat adipocyte tissue after administration of recombinant TNF-α, thus increasing circulating FFA levels [10]. Zhang *et al.* in his experimental study concluded that TNF-α was shown to stimulate lipolysis in human healthy adipocyte tissue [11]

The role of FFA to endothelial function in RA patients is not known, but in a study by Ormseth *et al.* (2013) on Systemic Lupus Erythematosus (SLE) patients, there was an association between FFA and endothelial activation markers *i.e.* E-selectin (r = 0.33, P = <0.001) and ICAM-1 (r = 0.35, P <0.001), but there is no correlation with sVCAM-1 [12].

These theories then raise the question of whether the process of endothelial dysfunction and atherosclerosis in RA patients can occur through TNF-α stimulation mechanisms against lipolysis.

## 2 METHODS

This research was a cross sectional design research conducted at Rheumatology Clinic, Cipto Mangunkusumo Hospital from October 8^th^ 2017 until November 15^th^ 2017. The subjects involved are rheumatoid arthritis patients who seek treatment at Rheumatology Clinic, Department of Internal Medicine and met the inclusion criterias: 1. Patients who have been diagnosed rheumatoid arthritis according to ACR / EULAR 2010 criteria. 2. Age ≥ 18 years. 3. Willing to be included in research and sign the informed consent form as research subject.

Patients with acute infection, diabetes mellitus, hypertension, dyslipidemia or obesity, suffer from cardiovascular disorders, such as coronary heart disease, heart failure, arrhythmia, or other vascular problems, such as stroke; has a smoking habit or previous smoking history; suffer from other autoimmune diseases besides rheumatoid arthritis; suffer from osteoarthritis accompanied by genus effusion; were in exclusion of this study. Subjects who met the inclusion criteria were chosen consecutively as many as 35 people according to the large sample calculation using the reference correlation coefficient from Klimiuk *et al.* [13]. The subjects then underwent serum sampling for laboratory tests of TNF-α, FFA, and sVCAM-1 levels. TNF-α examination was performed using the Quantikine ELISA (Human TNF-α Immunoassay) kit from R & D Systems Incorporate Minneapolis, Minnesota, USA. VCAM-1 examination was performed using the ELISA Quanticine kit (Human sVCAM-1 / CD106 Immunoassay) from R & D Systems Incorporate Minneapolis, Minnesota, USA. While the FFA examination as performed using the kit Free Fatty Acid Quantification Colorimetric / Fluorometric from BioVision Incorporate Milpitas, California, USA.

The data was correlated using Pearson analysis, or alternatively Spearman analysis. [14, 15].

The sudy had been approved by the committee of the Medical Research Ethics of the Faculty of Medicine, Universitas Indonesia.

## 3 RESULTS

### 3.1 Basic Characteristics

Most of the subjects of this study were women (97.1%) with an average age of 45.29 years. Most patients had moderate disease activity (65.7%). The full characteristics of the subject are shown in Table **1**. Subject characteristics based on levels of inflammatory mediator and adhesion molecule are shown in Table **2**.

### 3.2 Correlation Between TNF-α Level and sVCAM-1 Level in Rheumatoid Arthritis

**Fig. (1):**
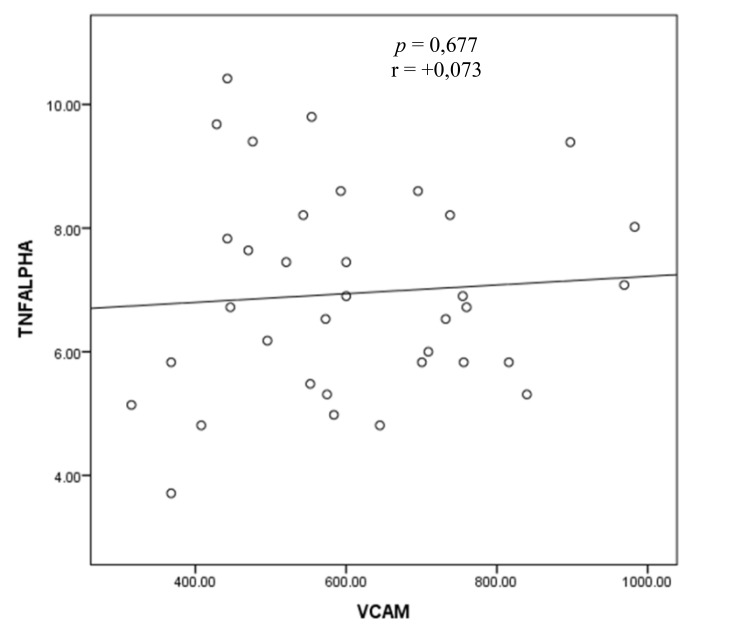
Scatter plot of correlation between TNF-α level and sVCAM-1 level in Rheumatoid Arthritis.

The result of bivariate test on the correlation between TNF-α and sVCAM-1 levels with Pearson correlation test showed that r = 0.073 (p = 0.677) (Fig. **1**).

Based on these results, there appears to be no correlation between TNF-α levels and sVCAM-1 levels.

### 3.3 Correlation between FFA Level and sVCAM-1 Level in Rheumatoid Arthritis

The bivariate analysis test to assess the correlation between the FFA and sVCAM-1 variables was performed by Pearson test and shown in Fig. (**2**).

The correlation between the FFA and sVCAM-1 variables as illustrated in Fig. (**2**) shows a significant correlation with the direction of negative correlation and weak correlation strength.

### 3.4 Correlation Between TNF-α Level and FFA Level in Rheumatoid Arthritis

The bivariate analysis test to assess the correlation between the TNF-α and FFA variables was performed by Pearson test and shown in Fig. (**3**).

Fig. (**3**) shows the correlation between and FFA with negative direction and weak correlation strength but these results show a non-significant relationship.

## 4 DISCUSSION

### 4.1 Characteristics of Subjects

The majority of the subjects in this study were women (97.1%), which shows the ratio of female and male of this study is 40:1. This result was clearly different from those mentioned in many literatures, where RA ratio in the population among female is compared to male of 3:1 [16].

The average age of this research was 45.29 years. This average corresponds to the existing literature, which states that RA peak incidence occurs between the fourth and fifth decades[17]. The Foster *et al.* study showed a slightly different case, where the median age of the subject was 58.5 years [8].

All subjects in this study used MTX therapy, with or without steroids. The average dose of steroids used by the study population was 8 mg per day metilprednisolone. This therapy is in line with the EULAR recommendation that recommends MTX treatment as first-line therapy in RA[18].

### 4.2 TNF-α Level, sVCAM-1 Level, and FFA Level in Rheumatoid Arthritis Patients

The mean of TNF-α level in this study was 6.95 (± 1.65 pg / ml). The Foster *et al.* study conducted a study assessing levels of inflammatory mediators in RA patients with metabolic risk factors and obtained TNF-α levels of 5.9 (0-47.5) pg / ml.

In addition to TNF-α levels, differences were also found in sVCAM-1 levels. The mean of sVCAM-1 concentration in this research was 609.96 (±171.70) pg / ml. In the Foster study, obtained sVCAM-1 levels in the RA patient group of 439 (196-553) pg / ml [8].

Other parameters assessed in the Foster study were the levels of the ESR, which obtained a median of 23 (8-50) mm/hour [8]. This result was much different from that obtained in this study that the median of 42 (10 - 130). mm/hour Based on the differences in these levels, our study showed a higher degree of inflammation and disease activity than Foster's study.

The degree of disease activity was likely to be the cause of higher levels of TNF-α and sVCAM-1 levels in this study than the Foster’s study. In addition, other factors that may also play a role was the treatment used by the subject of the research. In the Foster’s study the majority of subjects (87%) used anti-TNF-α therapy, while in this study all patients only used MTX without any biologic agents [13].

In addition to therapeutic factors, patient compliance rates in treatment as well as duration of treatment were also factors that can not be excluded. In this study, the duration of illness was shorter with median 48 (3-300) months, while Foster’s study showed a longer time of median of 10.7 years. This is related to the control of the disease which is thought to be better in the Foster’s research subject.

The FFA concentration in this study was obtained a mean of 0.051 (±0.027 pg / ml), where the mean was equivalent to 2 x 10^-9^ mmol / l (±9.59 x 10^-11^ mmol / l). In the Ormseth study, FFA levels were obtained with a median of 0.56 mmol / L (0.383-0.748 mmol / L) [12]. 

Ormseth's study obtained an elevation of FFA serum in RA patients with metabolic syndrome (0.62 mmol / L [0.44-0.80]) compared with subjects without metabolic syndrome (0.54 mmol / L [0.37-0.69]) [12]. This result showed the role of metabolic disturbance factors as one of the factors affecting serum FFA levels. This is also thought to be the cause of low levels of FFA in this study, where there was no metabolic disorders in the study subjects.

### 4.3 Correlation Between TNF-α Level and sVCAM-1 Level in Rheumatoid Arthritis

The result of Pearson correlation test between TNF-α and sVCAM-1 levels was found to be a non-significant correlation. These results differ from those obtained in the Foster *et al*. study, wherein the correlation between TNF-α levels and sVCAM-1 levels showed significant results [8].

In the Foster’s study there was no exclusion of traditional cardiovascular risk factors such as DM, hypertension, smoking, obesity and dyslipidemia. Diabetes mellitus as well as other metabolic disorders can increase FFA levels thus affecting inflammation and oxidative stress in endothelium [18].

Among the types of FFA, Saturated Fatty Acid (SFA) has an important role in cardiovascular events (pro inflammation), while Unsaturated Fatty Acid (UFA) is more protective (anti-inflammatory) [18]. Study by Platat *et al.* demonstrated that compared adults with overweight, normal-weight adults were shown to have higher UFA levels (p <0.001) and lower SFA (p <0.001) [19]. The controlled metabolic risk factor in this study was also thought to be related to a better lipid profile, in which the fraction UFA was more dominant in this research subject. The effect of UFA as anti-inflammatory occurs through the mechanism of inhibition of the NF-kB pathway, where TNF-α-stimulated sVCAM-1 expression is proven to occur through transcription and protein translation, involving multiple pathways, one of which is the NF-kB [20, 21]. In addition, MTX is also thought to be another factor contributing to the inhibition of the NF-kB pathway, which, in an experimental study by Majumdar *et al.*, it was proved that there were inhibition of NF-kB pathway after administration of MTX 10μΜ and incubation for 60-120 min [22].

### 4.4 Correlation Between FFA Level and sVCAM-1 Level in Rheumatoid Arthritis

There was a significant correlation with the direction of negative correlation and weak correlation strength between FFA and sVCAM-1. Negative correlation means the higher the FFA level, the lower the sVCAM-1 level.The negative correlation in this study was allegedly influenced by the anti-inflammatory effect possessed by FFA, precisely the UFA fraction. In the study of Carluccio *et al.* oleic acid, one type of UFA, was shown to decrease sVCAM-1 expression by more than 40% after incubation for 72 hours on HUVEC media. From this study, it was thought that the effect of inhibition on the expression of adhesion molecules by oleates occurs through the mechanism of inhibition of Nuclear factor-kappa B (NF-kB) pathway [23].

The above points show another effect possessed by FFA aside from being a pro inflammatory compound, *vice versa*, as anti-inflammatory. The dominantly anti-inflammatory FFA fraction that present in this study is thought to be one of the causes of the negative correlation found in this study.

### 4.5 Correlation Between TNF-α Level and FFA Level in Rheumatoid Arthritis

Pearson correlation test results in this study showed no correlation between levels of TNF-α and FFA. The results were similar to the one that obtained in the Ormseth *et al.* (2013) study, in which the study also found no correlation between TNF-α and FFA levels (r = -0.002, p = 0.98) [12]. However, this was not compatible with the clinical evidence described earlier in this paper, which has shown the increased of intracellular lipolysis of mouse adipocyte tissue after the administration of recombinant TNF-α, thus increasing circulating FFA levels [10].

In Ormseth's study the results were thought to caused by chronic exposure to inflammatory cytokines that are likely to alter lypolytic activity, thereby impacting the effects of TNF-α stimulation on FFA and negative correlations between them. [12]. In addition, another possible underlying reason for the absence of a correlation between TNF-α and FFA is the anti-inflammatory properties of the dominant FFA fraction in the subject, so that the FFA examination method could not be generalized and should be done separately according to the specific fractions to meet the desired effect.

### 4.6 Strengths and Limitations

The strenght of this study is one of the studies in Indonesia that assessed the correlation between TNF-α levels, FFA levels, and sVCAM-1 levels in rheumatoid arthritis patients by excluding risk factors for metabolic disorders. The main limitation of this research is the cross sectional design in this study which cannot explain the causal relationship between the variables assessed.

## CONCLUSION

In conclusion, our study showed that there was a negative correlation between FFA levels and sVCAM-1 levels in RA patients. It may indicate another effect possessed by FFA that is protective against endothelial dysfunction. However, research is needed with better designs that can prove the cause-and-effect relationship. Besides, the examination of FFA should be done specificallyby means of a device capable of indicating specific types or fractions of FFA present in the sample, in order to demonstrate the specific effects posed by those fractions.

## Figures and Tables

**Fig. (2):**
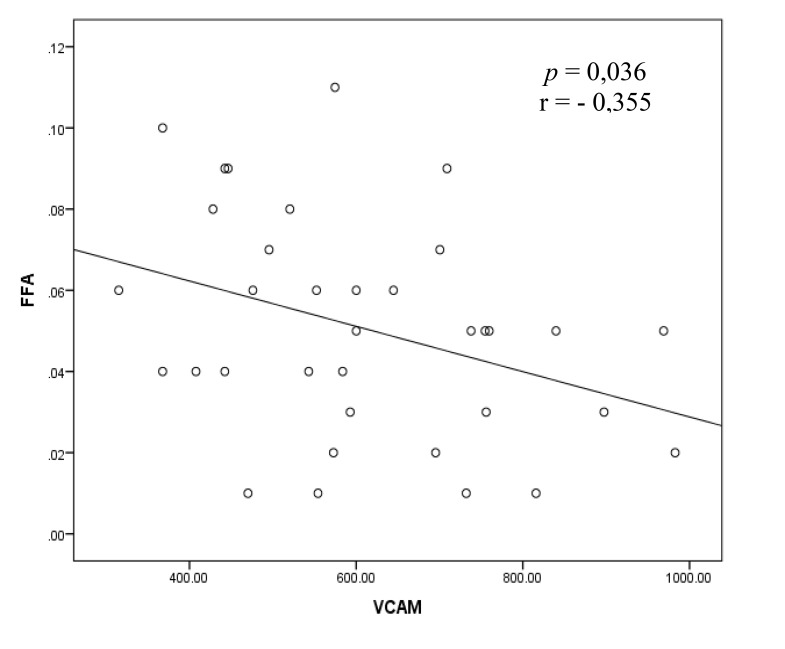
Scatter Plot of Correlation between FFA level and sVCAM-1 level in Rheumatoid Arthritis.

**Fig. (3):**
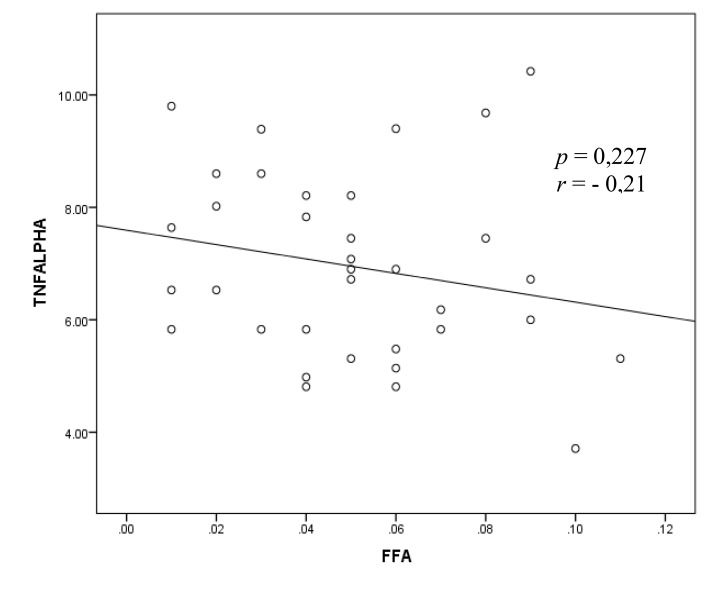
Scatter Plot of Correlation between TNF-α level and FFA level in Rheumatoid Arthritis.

**Table 1:** Demographic characteristic of the study.

Variable	*n =35*
***Sex***	
*Male*	*1 (2,9%)*
*Female*	*34 (97,1%)*
***Age (years)***	
*(mean, SD)*	*45,29 (±13,47)*
*Duration of illness (months), median(min-max)*	*48 (3 – 300)*
***Treatment***	
* MTX, n (%)*	* 3 (8,57%)*
*MTX + steroid, n (%)*	* 32 (91,42%)*
*ESR*	*42 (10 – 130)*
***DAS28ESR Score***	
* Median (min-max)*	*4,21 (2,01-8,40)*
* Remission, n (%)*	*2 (5,7)*
*Low, n (%)*	*5 (14,3)*
*Moderate, n (%)*	*23 (65,7)*
*High, n (%)*	*5 (14,3)*

**Table 2:** Subject characteristics based on levels of inflammatory mediator and adhesion molecule.

*Variable*	*Level*
*TNF-α (pg/ml), mean(SD)*	*6,95 (±1,65) pg/ml*
*FFA (pg/ml), mean(SD)*	*0,051 (±0,027) pg/m*
*sVCAM-1(pg/ml), mean(SD)*	*l609,96 (±171,70) pg/ml*
